# Expression Patterns of the *Drosophila* Neuropeptide CCHamide-2 and Its Receptor May Suggest Hormonal Signaling from the Gut to the Brain

**DOI:** 10.1371/journal.pone.0076131

**Published:** 2013-10-02

**Authors:** Shizhong Li, Teresa Torre-Muruzabal, Karen C. Søgaard, Guilin R. Ren, Frank Hauser, Signe M. Engelsen, Mads D. Pødenphanth, Annick Desjardins, Cornelis J. P. Grimmelikhuijzen

**Affiliations:** Center for Functional and Comparative Insect Genomics, Department of Biology, University of Copenhagen, Universitetsparken 15, Copenhagen, Denmark; Yale School of Medicine, United States of America

## Abstract

The insect neuropeptides CCHamide-1 and -2 are recently discovered peptides that probably occur in all arthropods. Here, we used immunocytochemistry, *in situ* hybridization, and quantitative PCR (qPCR), to localize the two peptides in the fruitfly *Drosophila melanogaster*. We found that CCHamide-1 and -2 were localized in endocrine cells of the midgut of larvae and adult flies. These endocrine cells had the appearance of sensory cells, projecting processes close to or into the gut lumen. In addition, CCHamide-2 was also localized in about forty neurons in the brain hemispheres and ventral nerve cord of larvae. Using qPCR we found high expression of the CCHamide-2 gene in the larval gut and very low expression of its receptor gene, while in the larval brain we found low expression of CCHamide-2 and very high expression of its receptor. These expression patterns suggest the following model: Endocrine CCHamide-2 cells in the gut sense the quality of food components in the gut lumen and transmit this information to the brain by releasing CCHamide-2 into the circulation; subsequently, after binding to its brain receptors, CCHamides-2 induces an altered feeding behavior in the animal and possibly other homeostatic adaptations.

## Introduction

The insect CCHamides are neuropeptides that have recently been discovered, but how this discovery initially took place is still a mystery. CCHamide was first mentioned by Zdárek et al. in 2000 as a “synthetic peptide, CCM” that had a weak stimulatory action on parturition of the tsetse fly *Glossina morsitans* (table 2 of [1]). Why this CCM peptide, which has the structure of a CCHamide, had been synthesized has remained unclear, since also a subsequent inquiry to the original authors [1] by other scientists in the field [2] did not resolve this issue.

In 2008, Roller and co-workers used the CCM sequence (GCLSYGHSCWGAHamide) in a TBLASTN homology search of the silkworm *Bombyx mori* genome sequence and that of three other insect genomes and found that these insects contained one or two CCHamide genes [2]. They also carried out *in situ* hybridization in *B. mori* and found expression of a CCHamide gene in several cells (probably neurons) in the brain and in several cells (probably endocrine cells) in the gut of the silkworm [2]. In 2010 Hansen and coworkers [3] showed, after having carried out more extensive TBLASTN searches, that all insects with a sequenced genome produce two CCHamides: CCHamide-1 (hallmark sequence: SCHSYGHSCWGAHamide), and CCHamide-2 (hallmark sequence: GCQ[or A, or S]AFGHSCY[or F]GGHamide). They also found that these two peptides are coded for by two separate genes. These results implicate that the CCM peptide originally synthesized in connection with experiments in the tsetse fly [1] was a CCHamide-1 peptide. Arthropods other than insects, however, such as crustaceans (e.g. the waterflea *Daphnia pulex*) and chelicerates (e.g. the tick *Ixodes scapularis*), contain only one gene coding for one CCHamide peptide, which has a structure that could not be clearly assigned to as either CCHamide-1 or -2 [3].

Hansen and coworkers [3] also cloned and identified two G protein-coupled receptors (GPCRs) from the fruitfly *Drosophila melanogaster* that were specifically activated by the predicted *D. melanogaster* CCHamide-1 peptide (sequence: SCLEYGHSCWGAHamide) or the predicted *D. melanogaster* CCHamide-2 peptide (sequence GCQAYGHVCYGGHamide). Shortly afterwards, in the beginning of 2011, the two *D. melanogaster* CCHamides were chemically isolated from the midgut of *D. melanogaster* larvae and adult flies and subsequently sequenced by mass spectrometry [4]. This structural work confirmed the sequences of the two *D. melanogaster* CCHamides and, also, that the two cysteine residues formed a cystine bridge, thereby making the peptides cyclic [4]. Both the structures of the two *D. melanogaster* CCHamides and the identities of their specific receptors were confirmed in December 2012 by Ida and co-workers [5].

Not much is known about the biological actions of the two *D. melanogaster* CCHamides and where in the fruit fly these two peptides and their receptors are produced. In the present paper we therefore localized the two CCHamides using immunocytochemistry, *in situ* hybridization, and quantitative PCR (qPCR). We also used qPCR to localize the two CCHamide receptors and to monitor the expression of the two peptides and their receptors in various developmental stages of the fruitfly.

## Materials and Methods

The *D. melanogaster* strain Canton S was used. These flies were cultured as described in [6].

qPCR was carried out from different developmental stages and different body parts of male and female *D. melanogaster*. Total RNA was isolated from these intact animals and organs using the RNeasy mini kit (Qiagen, Hilden, Germany), and cDNA was synthesized as described in [6]. qPCR was performed using the Brilliant II SYBR Green Master Mix (Agilent) and a MX3000P qPCR machine [6]. The other qPCR conditions, programs and subsequent calculations were carried out as described in [6].

We used three different reference genes for our qPCR: RNApolIII, RpL32 and RpL11. Their qPCR primer sequences as well as the primer sequences for the two CCHamide and the two receptor genes are given in Table S1. Reference gene stability was calculated as in [6] and was within acceptable limits for heterogeneous samples: M = 0.942 and CV = 0.364. For definitions of these reference gene stability factors M and CV see 7.

The qPCR experiments have been carried out 2-3 times (including independent tissue sampling, mRNA isolation, etc.) with very similar results. Statistical analysis was performed using a one-way analysis of variance (ANOVA) followed by Newman-Keuls post hoc tests for pair-wise comparisons.

*** means P < 0.001.

*In situ* hybridization was carried out as described in the Supporting Information and in Table S2.

Antisera against *D. melanogaster* CCHamide-1 or CCHamide-2 were commercially obtained from Genemed Synthesis, San Antonio, USA. They were raised in four rabbits immunized with CCHamide-1 and in two rabbits immunized with CCHamide‑2, both antigens being coupled with glutaraldehyde via their N-termini to keyhole limpet hemocyanin. The antisera used in this study were G6590-bleed2 (against CCHamide-1) and G6272-bleed2 (against CCHamide 2).

The mouse monoclonal antibodies against synapsin (3C11, anti-SYNORF1-s), bruchpilot (nc82-s), and fasciclinII (ID4 anti-FasII-s) were obtained from Developmental Studies Hybridoma Bank (University of Iowa, USA).

For immunocytochemistry, the guts and brains (whole CNS, i.e. the two brain hemispheres and ventral nerve cord) from third instar larvae and the guts and brains (whole CNS) from adult male and female flies were fixed at 4°C overnight in 4% paraformaldehyde in PBS, containing 0.1% Triton X-100(PBT). The samples were subsequently washed 3 times 10 min in PBT, and then incubated for 30 min in PBT containing 5% bovine serum albumin. The samples were then transferred to rabbit CCHamide-1 antiserum (1:200 in PBT plus 5% BSA) or CCHamide-2 antiserum (1:2000 in PBT plus 5% BSA) and incubated for 22 h at 4°, after which the same washing procedure as above was carried out. The specimens were subsequently incubated with donkey anti-rabbit IgG coupled to Alexa Fluor 488 (Invitrogen) diluted 1:1500 in PBT, containing 5% BSA for 22 h at 4°C, after which a four times washing procedure was carried out as described above.

For double-labeling, using the rabbit CCHamide-2 antisera and the mouse monoclonal antibodies, the specimens were incubated in a mixture of rabbit antiserum 1:2000 diluted as described above and a 1:10 dilution of either synapsin or bruchpilot mouse monoclonal antibody (supernatant), or a 1:75 dilution of the fasciclinII mouse monoclonal antibody (supernatant). After the incubation and washing procedures as described above, the specimen were incubated with a mixture of donkey anti-rabbit IgG coupled to Alexa Fluor 488 diluted 1:1500 as described above and goat anti-mouse IgG coupled to Alexa Fluor 594 (Invitrogen) diluted 1:1000. The same incubation and washing procedures were used as for the single antigen labeling experiments.

In the beginning of our studies we mounted guts and brains without the help of small rings, but for later experiments we used special rings (Secure-Seal imaging spacers, Sigma) for mounting the larval brains to avoid compression of the brains and obtain better information of dorsal and ventral localizations of the various neurons.

The samples were inspected with a Zeiss Axio Observer A1 fluorescence microscope and photographed with an AxioCam ICM1, using ZEN software.

Controls included preimmune antisera and primary antisera preabsorbed either with CCHamide-1 or -2 (both at 2x10^-5^ M). These controls did not yield any staining. Furthermore, these controls showed that the CCHamide-2 antiserum has no (or only very low) affinity for CCHamide-1, because its staining was not reduced after pre-absorption with CCHamide-1.

## Results

### 1. Endocrine cells in the midgut produce the *D. melanogaster* CCHamides-1 and -2

**Figure 1:**
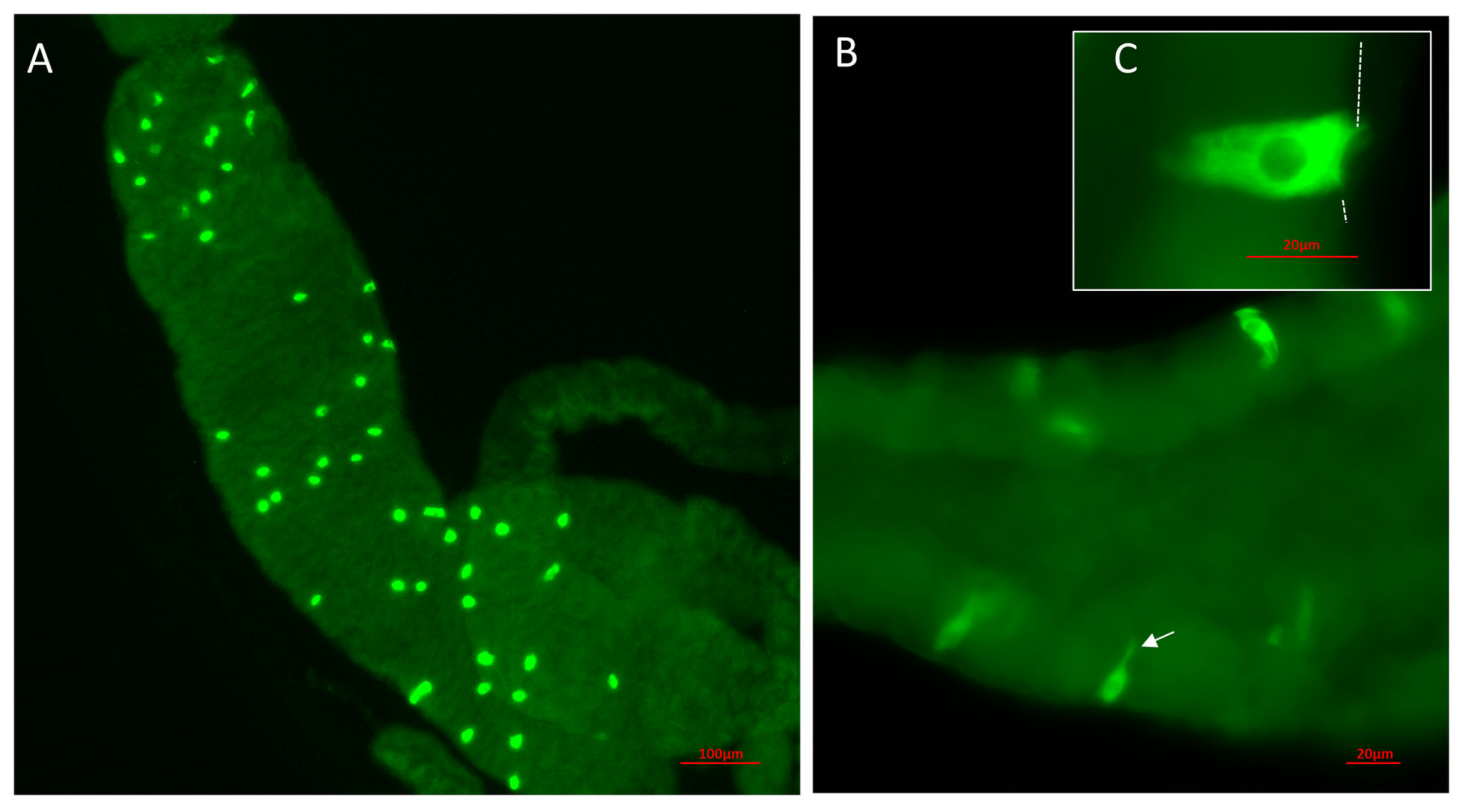
Endocrine cells in the body wall of the midgut of third instar *D. melanogaster* stained for CCHamide-2. A. An overview, showing numerous endocrine cells in the anterior part of the anterior midgut (see Figure 3 for a schematic drawing). Scale bar = 100 µm. B. A closer view of the endocrine cells, showing protrusions (arrow) directed toward the lumen of the gut. Scale bar = 20 µm. C. Close-up of a single endocrine cell. The basal part of the cell is in contact with the body circulation (the border of the gut epithelial cells and body cavity is stippled). Note that these cells are 25-40 µm in length. Scale bar = 20 µm.

**Figure 2:**
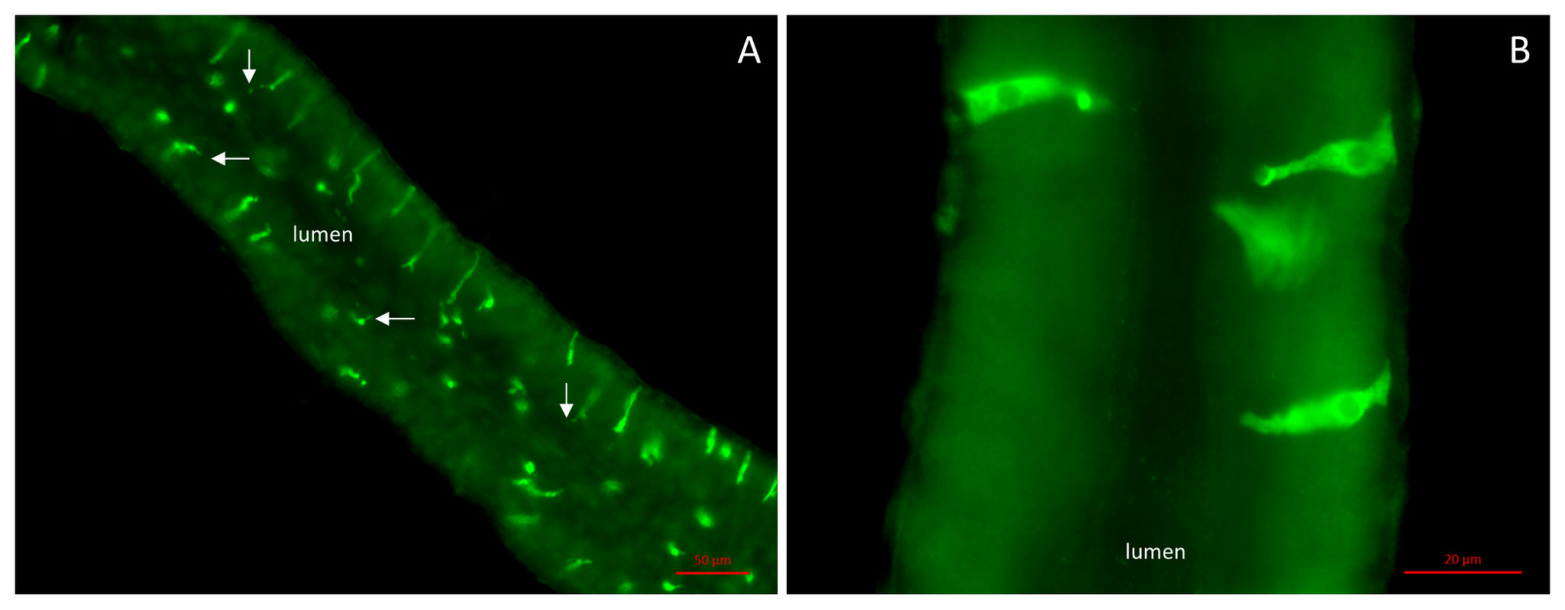
The anterior midgut of adult flies containing CCHamide-2 immunoreactive endocrine cells. (See figure 3 for a schematic drawing.) A. An overview of the anterior midgut showing the gut lumen and CCHamide-2 containing endocrine cells that project small hair-like processes (arrows) into the lumen. Note that these cells are 25-50 µm. Scale bar = 50 µm. B. Close up of the endocrine cells in the anterior midgut, showing that their bases are in contact with the body cavity and that their more slender apical parts project into the lumen. Scale bar = 20 µm.

We raised antisera against *D. melanogaster* CCHamide-1 (four rabbits immunized) and CCHamide-2 (two rabbits immunized) and found that the CCHamide-2 antisera were always much more potent than the CCHamide-1 antisera. The CCHamide-2 antisera stained strongly immunoreactive endocrine cells in the midgut of third instar larvae and in the midgut of adult male and female flies (Figure 1, Figure 2). In third instar larvae these endocrine cells had a triangular appearance with a broad basis directed towards the body cavity and a more slender part directed towards the gut lumen (Figure 1B, Figure 1C). In the midgut of adult flies these cells were more slender than in the larval gut (Figure 2A, Figure 2B), while the apical part, which was directed towards the gut lumen, had a small “knob” often containing a tiny process or hair-like protrusion possibly extending into the gut lumen (arrows in Figure 2A). Both in larvae and in adult flies these endocrine cells were quite large (25–40 µm) (Figure 1C, Figure 2).

**Figure 3:**
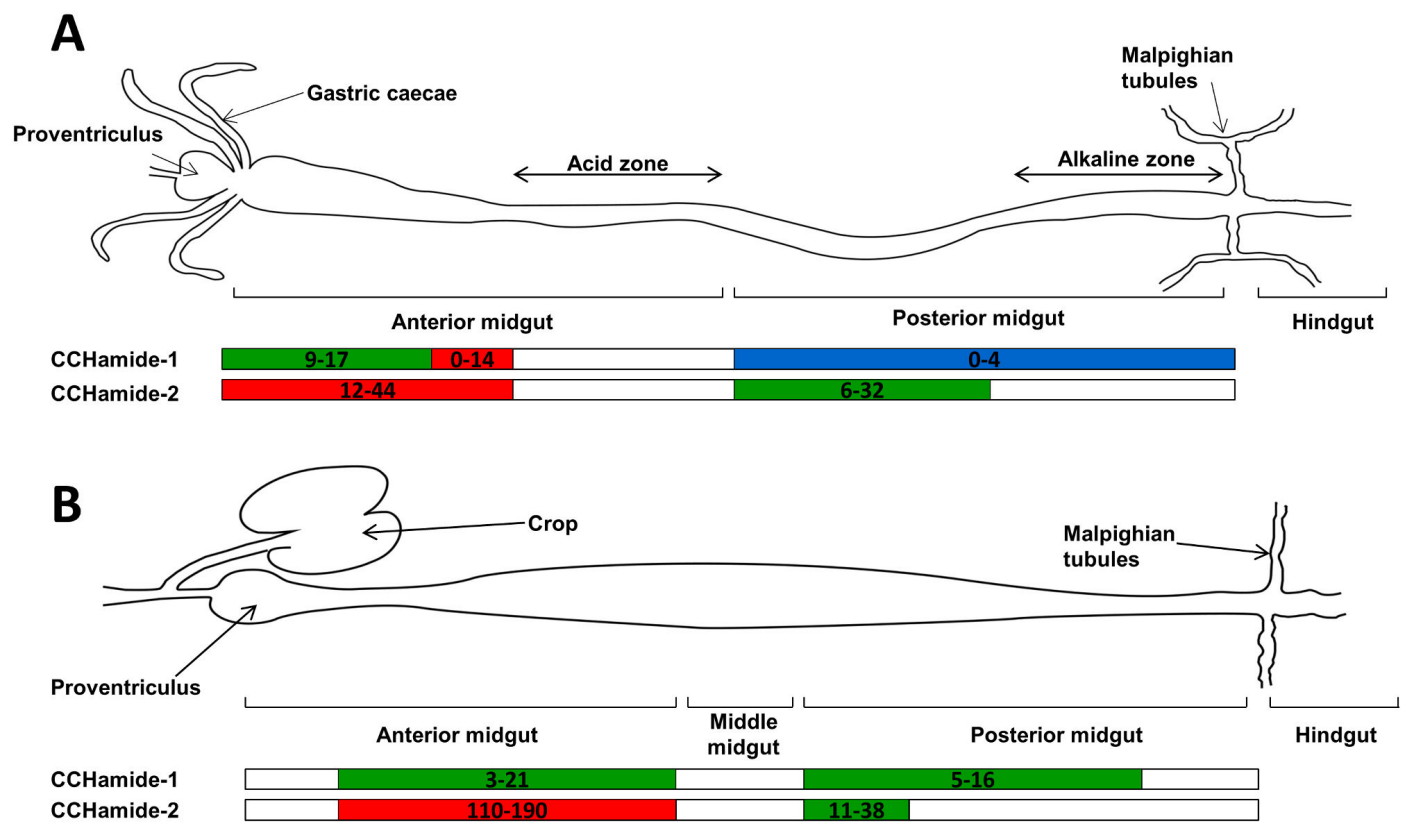
Schematic representations of the guts from third instar larvae and adult flies. The numbers give total numbers of counted CCHamide immunoreactive endocrine cells per midgut segment. These numbers vary from animal to animal and we have, therefore, given both the lowest and highest number counted in at least 50 animals. The colors indicate the concentrations of CCHamide immunostained cells from blue (low concentration) to green (intermediate) to red (high concentration). A. third instar larvae. Both the CCHamide-1 and -2 endocrine cells occur in the anterior midgut and in the anterior part of the posterior midgut. B. Adult male/female flies. The CCHamide-1 and -2 endocrine cells occur mainly in the anterior midgut and to a somewhat lesser extend in the posterior midgut.

The CCHamide-2 immunoreactive endocrine cells occurred mainly in the anterior part of the larval gut and to a smaller extend in the posterior part (Figure 3A). In the acid zone of the middle midgut and the alkaline zone of the posterior midgut, we did not find CCHamide-2 immunoreactive endocrine cells (Figure 3A). The situation in the adult midgut resembled that of the larvae (Figure 3B). Both in larval and adult flies we found that the number of CCHamide-2 cells in the gut was highly variable between different animals (Figure 3).

While two rabbits yielded excellent CCHamide-2 antisera (Figure 1, Figure 2), none of the four rabbits that were immunized with the CCHamide-1 antigen yielded antisera of comparable quality. Yet, we could reasonably well visualize CCHamide-1 immunoreactive endocrine cells in the *D. melanogaster* midgut and count them. We found that the CCHamide-1 endocrine cells had a distribution in the larval midgut that was somewhat different from the distribution of the CCHamide-2 cells. Especially in a small region of the posterior part of the anterior midgut just before the acid zone (Figure 3A), we found high concentrations of CCHamide-1 endocrine cells.

To confirm our immunocytochemical findings, we also carried out *in situ* hybridization, using probes specifically directed against either the CCHamide-1 or CCHamide-2 precursor mRNAs [3] (Figure S1, table S2, Supporting Information). These *in situ* hybridizations showed distributions of CCHamide-1 and CCHamide-2 cells that were similar to the ones shown in Figure 3 for staining using antisera.

### 2. Neurons in the larval central nervous system produce CCHamide-2

**Figure 4:**
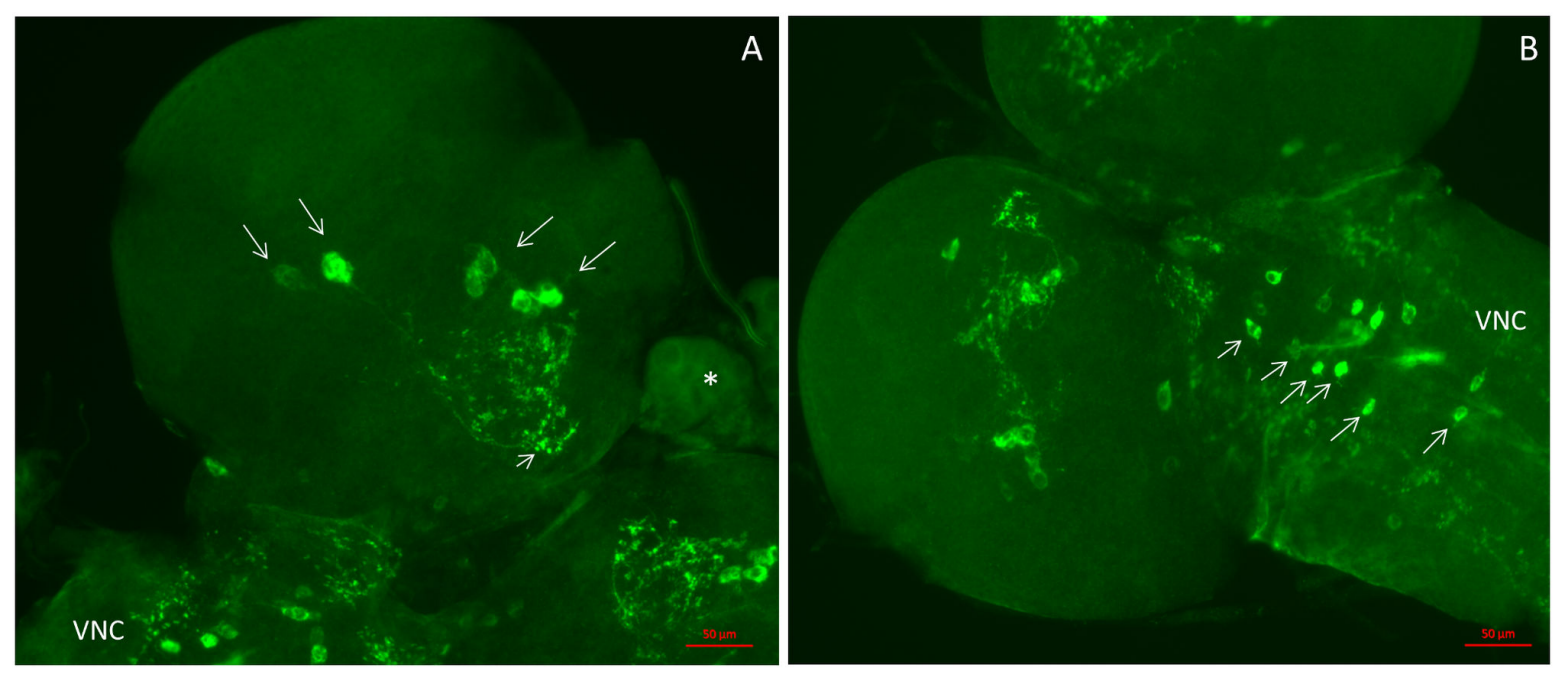
Neurons in the larval CNS stained with CCHamide-2 antisera. These preparations are somewhat compressed, because of mounting without the application of spacer rings. These spacer rings were present in the preparations shown in Figure 6 and Figure 7. For a schematic representation of the neurons and neuropil in the two larval brain hemispheres and ventral nerve cord, see Figure 5. A. Ventral view of one hemisphere of the brain, showing 7-8 immunoreactive neurons located in the central region (long arrows) and processes (neuropil) projecting to other regions of the brain (short arrow). This neuropil corresponds to the neuropil indicated by number 3 in Figure 5. A piece of the ring gland (asterisk) is also visible, as well as a piece of the ventral nerve cord (VNC). Scale bar = 50 µm. B. Ventral view of another brain hemisphere with the ventral nerve cord. At least 12 nerve cells (short arrows) can be seen here symmetrically ordered along the midline of the ventral nerve cord (VNC). Scale bar = 50 µm.

**Figure 5:**
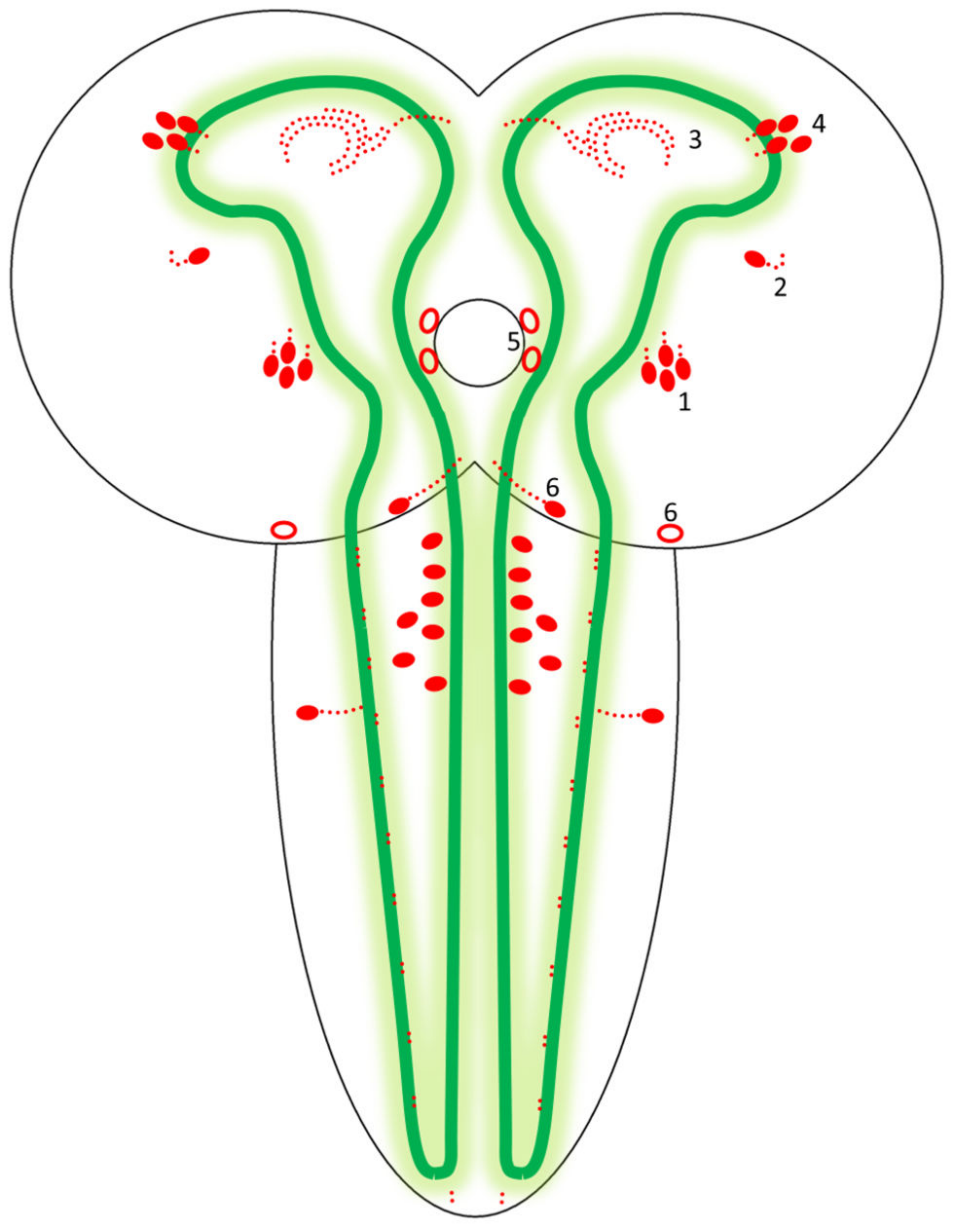
Schematic drawing of the localizations of the CCHamide-2 immunoreactive neurons and neuropil in the CNS of third instar larvae. The drawing shows the two hemispheres and the ventral nerve cord. The central neuropil of the larval CNS (stained with a synapsin mouse monoclonal antibody, cf. Figure 6 and Figure 7) is outlined by green lines and shades. Neuronal perikarya and neuropil are drawn in red. Weakly immunoreactive perikarya are drawn as open red symbols. The perikarya indicated by 1 and 2 in the right hemisphere are located dorsally in each hemisphere; the perikarya indicated by 4, and 5, and 6 are located in the ventral parts of the hemispheres. The neuropils indicated by 3 as red dots in the anterior parts of the central neuropil are located partially ventrally and partially medially between the levels of the neurons 1 and 4. All perikarya in the ventral nerve cord are located in the ventral part of this nerve cord. They belong to the three fused thoracic ganglia.

Another organ, where CCHamide-2 is produced is the larval central nervous system (CNS). Using CCHamide-2 antisera we found staining of about thirteen neurons in each hemisphere of the brain (Figure 4; Figure 5). These neurons can be grouped into 4 closely located perikarya that lie dorsally in the central part of each hemisphere (indicated by 1 in Figure 5), a single perikaryon that also lies dorsally (indicated by 2 in Figure 5), and 4 very closely located perikarya that lie ventrally in the more anterior part of each hemisphere (indicated by 4 in Figure 5). Furthermore, there are 2 weakly stained neurons bordering the esophagus (indicated by 5 in Figure 5) and 2 neurons in the posterior part of each hemisphere (indicated by 6 in Figure 5).

**Figure 6:**
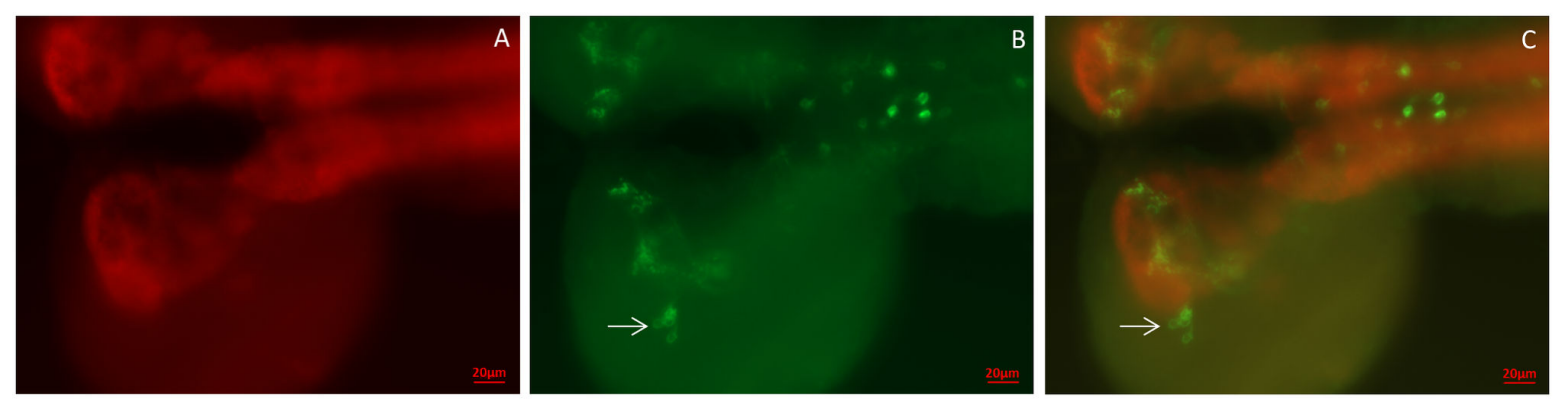
Double-labeling of the CNS of third instar larvae, using staining with a mouse monoclonal antibody against *Drosophila* synapsin (red fluorescence) and a rabbit antiserum against CCHamide-2 (green fluorescence). A. Dorsal view of the ventral plane of the CNS, showing the central neuropil in each hemisphere and in the anterior part of the ventral nerve cord. The excitation and emission wavelengths used were selective for Alexa Fluor 594 fluorescence (red). B. The same focal plane as in A, but now using excitation and emission wavelengths selective for Alexa Fluor 488 fluorescence (green). C. Merge of A and B. The CCHamide-2 immunoreactive neuropil is located in the anterior part of the central neuropil in each hemisphere. The arrow points to a group of CCHamide-2 immunoreactive perikarya (somewhat out of focus) belonging to the group 4 perikarya of Figure 5. Scale bar = 20 µm.

To better determine the locations of the perikarya and neuropil in Figure 5, we carried out double-labeling experiments, using our rabbit antiserum against CCHamide-2 and a mouse monoclonal antibody against *Drosophila* synapsin, which visualizes neuropil (Figure 5, Figure 6). These experiments showed that processes extended from neurons 1, 2, and 4 into the anterior portion of the central neuropil of the two hemispheres (indicated by 3 in Figure 5).

**Figure 7:**
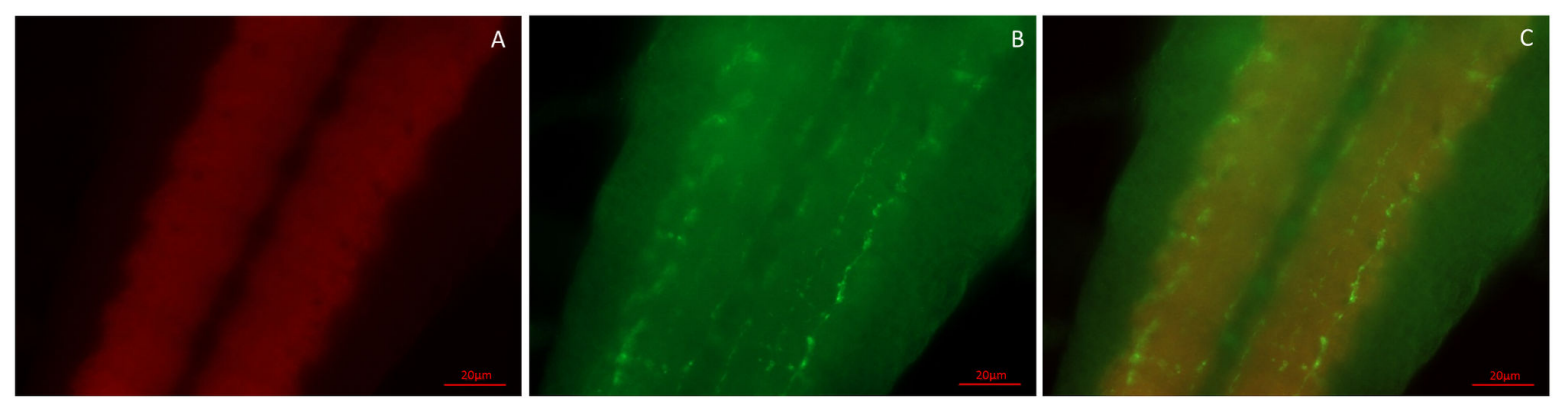
A double-labeling experiment as in Figure 6, but now showing the posterior part of the ventral nerve cord. A. A dorsal view of the dorsal plane of the ventral nerve cord stained with the synapsin monoclonal antibody, showing the two columns of neuropil of the ventral nerve cord (red). B. The same focal plane as in A, but now showing staining with the CCHamide-2 antiserum (green). C. Merge of A and B. Dorsally located CCHamide-2 immunoreactive processes can be seen projecting in a longitudinal orientation over the whole nerve cord. Scale bar = 20 µm.

The anterior part of the ventral nerve cord contained sixteen CCHamide-2 immunoreactive neurons ordered symmetrically with respect to the ventral nerve cord midline (Figure 4B, Figure 5). Double-labeling experiments, using our rabbit CCHamide-2 antiserum and a mouse monoclonal antibody against the *Drosophila* protein fasciclinII, which, among other structures, outlines the three fused thoracic ganglia, showed that the neuronal perikarya in the ventral nerve cord were all located in the three thoracic segments (Figure 5). While the perikarya and some of their processes were located in the ventral part of the ventral nerve cord, we also found longitudinal oriented processes in the dorsal part of the ventral nerve cord (Figure 7).

CCHamide-1 antisera did not yield any staining of the larval CNS. Similarly, in the brain of adult flies we did not find immunostaining for either CCHamide-1 or -2.

### 3. Quantitative PCR (qPCR) of CCHamide-1 and -2, and CCHamide-1 and -2 receptor mRNAs

**Figure 8:**
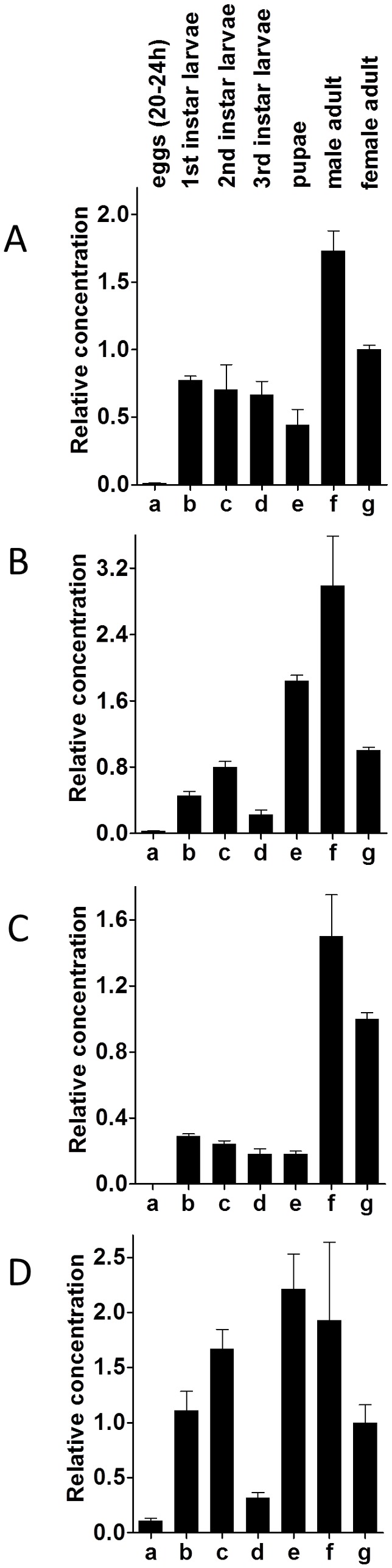
qPCR data for the expression of the *D. melanogaster* CCHamide-1 gene (CG14358), the CCHamide-1 receptor gene (CG14593), the CCHamide-2 gene (CG14375), and the CCHamide-2 receptor gene (CG30106), in different developmental stages. Embryos, 20-24 h after egg laying (a); first instar larvae, 48 h after egg laying (b); second instar larvae, 72 h after egg laying (c); third instar larvae, 96 h after egg laying (d); pupae, 7 d after egg laying (e); and adult male (f) and female (g) flies both 10 d after egg laying, or 1 d after eclosion. At least 25 animals were pooled from each developmental stage. These pools are the same in (A)-(D). The mRNA concentrations in each panel are given relative to column g (g=1). The qPCR experiments were run as triplicates. The vertical bars in each column (which are sometimes smaller than the lines of the column) represent SEM. A. Expression of the *D. melanogaster* CCHamide-1 gene in the different developmental stages. B. Expression of the *D. melanogaster* CCHamide-1 receptor gene. C. Expression of the CCHamide-2 gene. D. Expression of the CCHamide-2 receptor gene.

We carried out qPCR on different developmental stages of *D. melanogaster* (Figure 8). We found that the two peptide genes and the two receptor genes were only very lowly expressed in eggs. This expression increased in the three larval stages and pupae and in adult male and female flies (Figure 8).

**Figure 9:**
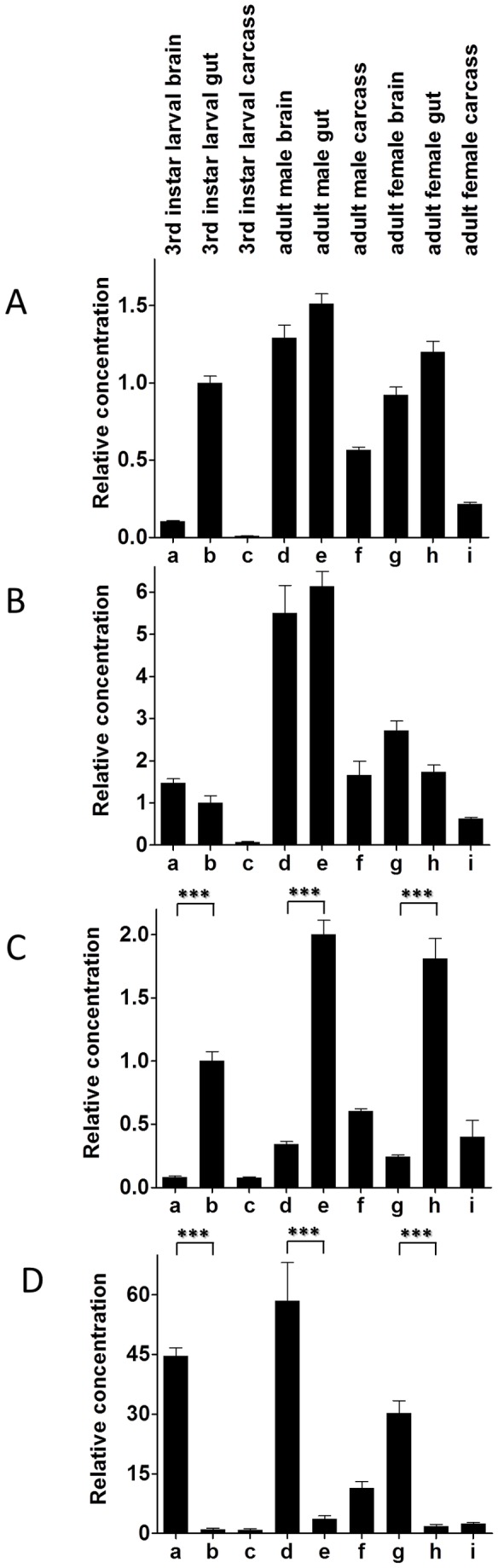
qPCR data for the expression of the *D. melanogaster* CCHamide-1 gene (CG14358), the CCHamide-1 receptor gene (CG14593), the CCHamide-2 gene (CG14375), and CCHamide-2 receptor gene (CG30106) in different organs or body parts of larval and adult flies. Third instar larval brain (a); third instar larval gut (b); third instar larval carcass (= rest of the body without brain and gut) (c); adult male brain (d); adult male gut (e); adult male carcass (= rest of the body without brain and gut) (f); adult female brain (g); adult female gut (h); adult female carcass (= rest of the body without brain and gut) (i). At least 25 organs were pooled for each qPCR measurement. These pools are the same in (A)-(D). The mRNA concentrations in each panel are given relative to column b (b=1). Other conditions are as in Figure 8. A. Expression of the *D. melanogaster* CCHamide-1 gene. B. Expression of the *D. melanogaster* CCHamide-1 receptor gene. C. Expression of the CCHamide-2 gene. D. Expression of the CCHamide-2 receptor gene. Statistical analyses showed that there is a significant difference (P < 0.001) between the columns a and b; d and e; and g and h in Figure 9C and Figure 9D.

When we compared different body parts from third instar larvae and adult male and female flies, the most striking results were obtained from measuring CCHamide-2 and its receptor in third instar larvae (Figure 9C, a, b, c; Figure 9D, a, b, c): In the third larval instar brain, there was very little CCHamide-2 mRNA (Figure 9C, a), while there was a large amount of CCHamide-2 receptor mRNA (Figure 9D, a). In the larval gut it was just the opposite: The CCHamide-2 gene was very strongly expressed (Figure 9C, b), while the receptor gene showed very low expression (Figure 9D, b). These findings, which have been repeated in three independent qPCR experiments (including independent tissue sampling) with very similar results, suggest that CCHamide-2 is mainly produced in the gut endocrine cells, while its receptor is mainly produced in the brain.

Also in adult male and female flies the CCHamide-2 gene is strongly expressed in the gut (Figure 9C, e and h), while the receptor gene is only very lowly expressed in the gut (Figure 9D, e and h). Again, in the brain there is strong receptor gene expression (Figure 9D, d and g), while the peptide gene expression is low (Figure 9C, d and g).

## Discussion

*In situ* hybridization studies by other researchers have previously localized CCHamide mRNA in the CNS and gut of the silkworm *Bombyx mori* [2]. In our present study, using immunocytochemistry, we have for the first time localized CCHamide-2 peptides in endocrine cells of the gut of larval and adult *D. melanogaster* (Figure 1, Figure 2). These endocrine cells have a very characteristic shape: They have a broad basal part that is directed towards and in contact with the body cavity (Figure 2B) and a slender apical part, containing a knob (Figure 2B), which is bearing a hair-like extension that probably reaches into the lumen of the gut (Figure 2A). This morphology and orientation of the CCHamide-2 endocrine cells in the gut already suggest that the cell perceives information from the gut lumen (possibly food quality: amino acid/lipid/carbohydrate content) and that it signals this information from the gut to other organs in the fly body (for example to the brain) by releasing CCHamide-2 into the circulation.

**Figure 10:**
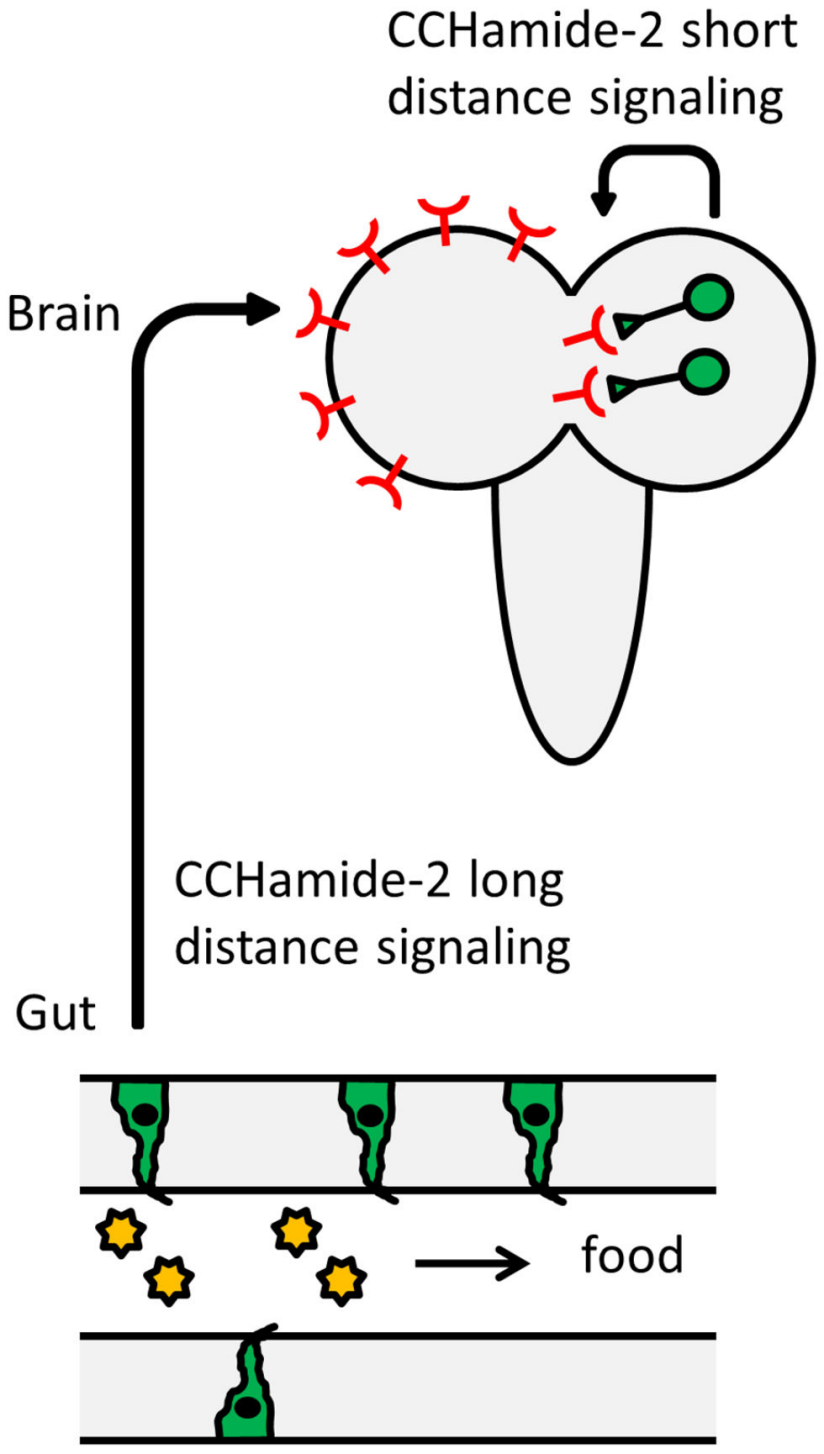
Cartoon of our proposed mechanism for the hormonal action of CCHamide-2 in third instar larvae. This hypothetical model is based on the sensory nature of the CCHamide-2 immunoreactive endocrine cells in the gut (Figure 1) and on our findings that there is abundant expression of the CCHamide-2 receptor gene in the brain (Figure 9D, a), while this expression is virtually absent in the gut (Figure 9D, b), combined with the high CCHamide-2 peptide gene expression in the gut (Figure 9C, b). This model predicts that the quality of the food in the gut lumen is sensed by the CCHamide-2 immunoreactive endocrine cells in the gut wall. These cells release the CCHamide-2 peptides into the circulation, after which they reach the brain and bind to the CCHamide-2 receptors. This binding starts a cascade that leads to an altered (adapted) feeding behavior of the animal. In addition to this long-distance hormonal CCHamide-2 signaling, there is a local (synaptic or paracrine) CCHamide-2 signaling in the larval CNS, possibly also associated with feeding.

When we measured the CCHamide-2 receptor gene expression in third instar larvae, using qPCR, we found hardly any receptor expression in the gut (Figure 9D, b). In contrast, there was a high expression of these receptors in the brain: 45x the concentration that was expressed in the gut (Figure 9D, a). For the CCHamide-2 peptide it was the other way around. There was 12x higher expression in the gut (Figure 9C, b) compared to the brain (Figure 9C, a). Thus, when normalized for the concentration of CCHamide-2 expression, there is 540x higher CCHamide-2 receptor expression in the brain compared to the gut. These very strong differences in the receptor expression may suggest that CCHamide-2 is released from the gut into the circulation and that it subsequently activates its receptors in the brain (Figure 10). We propose a similar signaling pathway in adult male and female flies, because also here the CCHamide-2 receptor is strongly expressed in the brain (Figure 9D, d and g), while it is very lowly expressed in the gut (Figure 9D, e and h). Again, in accordance with the model of Figure 10, there is very high expression of the CCHamide-2 gene in the gut (Figure 9C, e and h). We would like to stress that our model of Figure 10 is purely hypothetical and that we do not have additional experimental data that support it.

For CCHamide-1 there are no convincing arguments for signaling from the gut to the brain, because CCHamide-1 receptor expression in the gut is still considerable (Figure 9B, b and e and h). CCHamide-1 might, therefore, act locally.

Using immunocytochemistry, we also found about 40 neurons in the larval CNS that produced CCHamide-2 (Figure 4; Figure 5). Thus, in addition to the proposed long-distance signaling of CCHamide-2 from the gut to the brain, there must also be a short distance (local) CCHamide-2 signaling inside the CNS (Figure 10).

*Drosophila* is a highly promising model for studying feeding, satiety, carbohydrate homeostasis, life span, insulin signaling, and human diseases such as diabetes and obesity [8,9]. Several neuropeptides are known to control feeding in *Drosophila*, such as short neuropeptide F[10, 11], neuropeptide F[10, 11], allatostatin A [12], kinins [13] and sulfakinins [14]. The discovery of another candidate, CCHamide-2, to this collection of feeding neuropeptides is extremely important, because it brings us closer to a more complete understanding of feeding regulation in the fruitfly.

Park and Kwon recently found that endocrine cells of the *D. melanogaster* adult gut express gustatory receptors [15]. By doing co-localization studies they determined that some of these gustatory receptors, for example Gr43a, which functions as a fructose receptor [16], are co-localized with endocrine cells containing neuropeptide F, while other gustatory receptors are co-localized with endocrine cells containing kinin or diuretic hormone-31 (DH31) [15]. It is therefore quite feasible that also the CCHamide-2 immunoreactive endocrine cells (Figure 2) are expressing a gustatory receptor, which perceives information about the nutritional value of food in the gut lumen. We propose that this nutritional information by the CCHamide-2 endocrine cells is not used for local gut responses, such as peristalsis, because there is hardly any CCHamide-2 receptor present in the gut, including its musculature (Figure 9D, b). Instead, we suggest that this signal is transmitted to the brain (Figure 9C, b), where it alters feeding behavior (Figure 10).

## Supporting Information

Figure S1 ***In situ* hybridizations, showing expression of the CCHamide-1 and -2 genes in endocrine cells of the larval and adult midgut of *D. melanogaster*.** See Figure 3 for a schematic presentation of the distribution of CCHamide-1 and -2 immunoreactive endocrine cells in the midgut. A. CCHamide-1 cells (the arrows show some examples) in the posterior part of the larval anterior midgut. This region corresponds to the region highlighted in red in Figure 3A. Scale bar = 100 µm. B. CCHamide-1 cells (the arrows show some examples) in the adult anterior midgut. Scale bar = 100 µm. C. CCHamide-2 cells (the arrows show some examples) in the larval anterior midgut. Scale bar = 100 µm. D. CCHamide-2 cells (the arrows show some examples) in the adult anterior midgut. Scale bar = 100 µm.
(PDF)

Materials and Methods S1 **Materials and methods for Figure S1.**
(PDF)

Table S1 **Primer sequences used in qPCR.** GenBank accession no. CCHamide-1, NM_001104314; CCHamide-2, NM_142028; CCHamide-1 receptor, NM_137397; CCHamide-2 receptor, NM_136355; RNApolII, NM_057358.3; RpL32, NM_170460; RpL11, NM_057706.4.
(PDF)

Table S2 **Primers used for *in situ* hybridization.**
(PDF)
