# Supplementary material for: Expression Patterns of the Drosophila Neuropeptide CCHamide-2 and Its Receptor May Suggest Hormonal Signaling from the Gut to the Brain
Source: PLoS One. 2013 Oct 2;8(10):e76131. doi: 10.1371/journal.pone.0076131 (PMC3788761; doi:10.1371/journal.pone.0076131)
Supplement: Figure S1 — In situ hybridizations, showing expression of the CCHamide-1 and -2 genes in endocrine cells of the larval and adult midgut of D. melanogaster. See Figure 3 for a schematic presentation of the distribution of CCHamide-1 and -2 immunoreactive endocrine cells in the midgut. A. CCHamide-1 cells (the arrows show some examples) in the posterior part of the larval anterior midgut. This region corresponds to the region highlighted in red in Figure 3A. Scale bar = 100 µm. B. CCHamide-1 cells (the arrows show some examples) in the adult anterior midgut. Scale bar = 100 µm. C. CCHamide-2 cells (the arrows show some examples) in the larval anterior midgut. Scale bar = 100 µm. D. CCHamide-2 cells (the arrows show some examples) in the adult anterior midgut. Scale bar = 100 µm. (PDF) [file pone.0076131.s001.pdf]

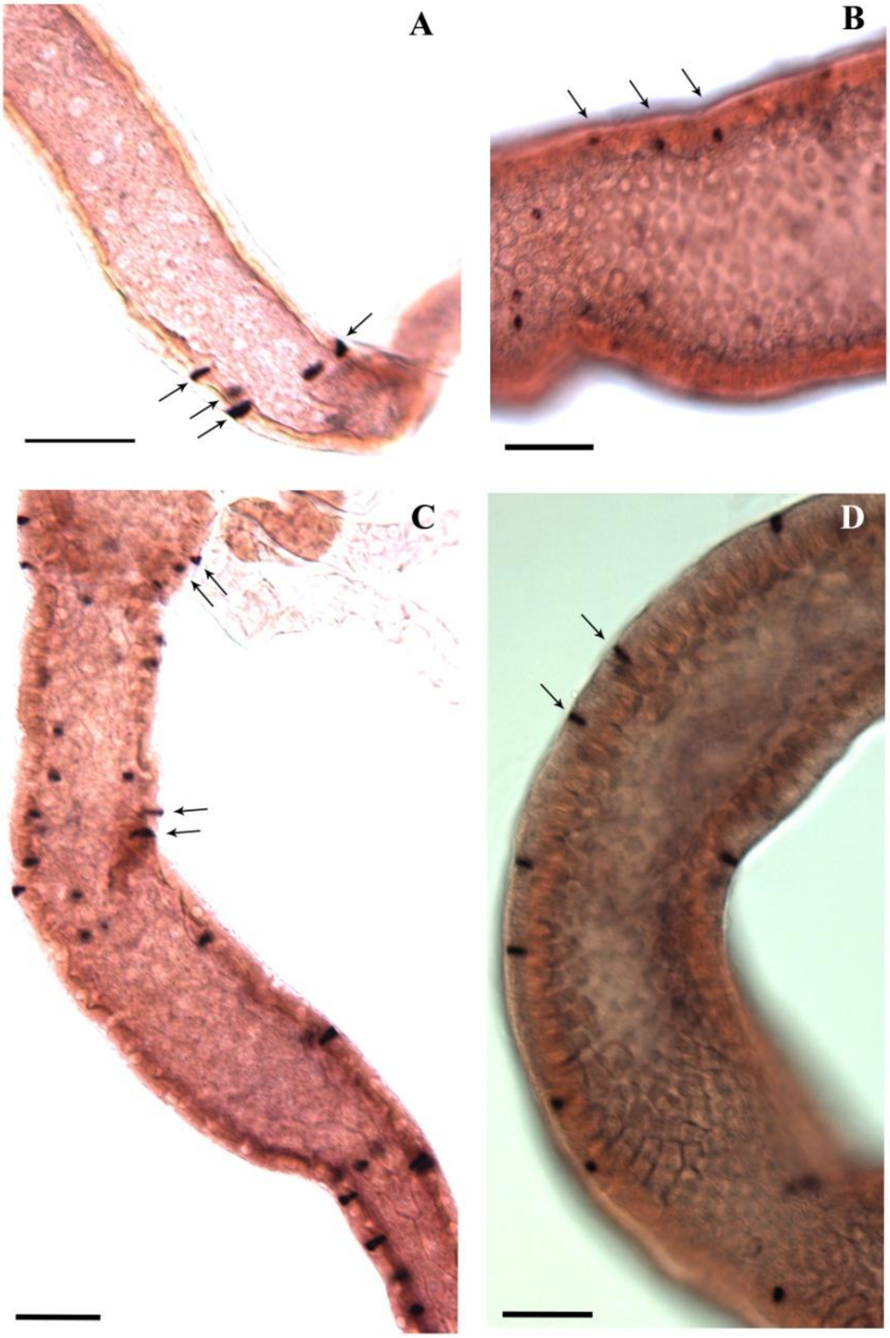

Fig. S1. *In situ* hybridizations, showing expression of the CCHamide-1 and -2 genes in endocrine cells of the larval and adult midgut of *D. melanogaster*. See Fig. 3 for a schematic presentation of the distribution of CCHamide-1 and -2 immunoreactive endocrine cells in the midgut. (A) CCHamide-1 cells (the arrows show some examples) in the posterior part of the larval anterior midgut. This region corresponds to the region highlighted in red in Fig. 3A. Scale bar = 100 µm. (B) CCHamide-1 cells (the arrows show some examples) in the adult anterior midgut. Scale bar = 100 µm. (C) CCHamide-2 cells (the arrows show some examples) in the larval anterior midgut. Scale bar = 100 µm. (D) CCHamide-2 cells (the arrows show some examples) in the adult anterior midgut. Scale bar = 100 µm.
