# Supplementary material for: Expression Patterns of the Drosophila Neuropeptide CCHamide-2 and Its Receptor May Suggest Hormonal Signaling from the Gut to the Brain
Source: PLoS One. 2013 Oct 2;8(10):e76131. doi: 10.1371/journal.pone.0076131 (PMC3788761; doi:10.1371/journal.pone.0076131)
Supplement: Materials and Methods S1 — Materials and methods for Figure S1. (PDF) [file pone.0076131.s002.pdf]

**Supplementary material**

## Materials and methods section for the *in situ* hybridization experiments shown in Fig. S1

To prepare Digoxigenin (DIG)-labeled RNA probes, nucleotides 301-839 of the CCHa1 and nucleotides 332-895 of the CCHa2 cDNA were PCR-amplified using primers shown in Table S2, cloned into pCR4-TOPO (Invitrogen) and sequenced. Plasmid DNA was linearized using NotI (antisense probe) or SpeI (sense probe) and purified by agarose gel electrophoresis. RNA probes were synthesized with the DIG RNA labeling kit (Roche). T3 RNA polymerase was used for the antisense probe, T7 RNA polymerase for the sense probe.

Tissues were fixed 20 min at room temperature in 4% paraformaldehyde in PBS, processed through methanol series diluted in PBST (PBS containing 0.1% Tween 20) with methanol:PBST ratios of 0:1, 3:7, 1:1, and 7:3, and finally washed 2 x 5 min in 100% methanol. Thereafter, tissue was rehydrated into PBST with methanol:PBST ratios of 7:3, 1:1, 3:7 and 0:1. Tissues were washed 5 x 5 min in PBST, treated 3 min with non-predigested Proteinase K (4 μg/ml in PBST), washed 4 x 5 min with PBST, refixed for 20 min in 4% PFA in PBS and washed 5 x 5 min in PBST.

Hybridization buffer (HB) contained 50% formamide, 5xSSC, 100 μg/ml salmon sperm DNA, 50 μg/ml heparin and 0.1% Tween 20. Tissues were washed 5 min in 1:1 HB:PBST, 5 min in 100% HB and prehybridized for two hours at 55 °C in 200 μl HB. RNA-probes were heated to 80 °C for 5 min and chilled on ice. After removal of HB from the tissues, 1 μl DIG-labeled RNA probe was diluted in 99 μl HB, added to the tissues and hybridized overnight.

After hybridization, the tissues were washed 5 x 1 hour at 55 °C with 200 μl HB, 5 min in 1:1 HB:PBST and 5 x 5 min in PBST. For staining, the tissues were incubated for one hour with MABT (2% Blocking Reagent for Nucleic Acid Hybridization and Detection (Roche) in 100mM maleic acid and 150 mM NaCl, pH 7.5) and for one hour with 10% goat serum in MABT.

Tissues were incubated overnight at 4°C with anti-DIG-AP fab fragments diluted 1:2000 in MABT. Prior to incubation, the diluted fab fragments were preabsorbed with fixed *D. melanogaster* overnight at 4°C. The day after antibody incubation, samples were washed 5 x 5 min with PBST. Tissue was stained using 2% NBT/BCIP in 0.1M NaCl , 0.1M Tris-HCl (pH 9). The staining reaction was stopped by washing 6 x 5 min with PBST. Stained tissue was mounted in 8:2 glycerol:PBS and observed under a microscope.
