# Supplementary material for: Expression Patterns of the Drosophila Neuropeptide CCHamide-2 and Its Receptor May Suggest Hormonal Signaling from the Gut to the Brain
Source: PLoS One. 2013 Oct 2;8(10):e76131. doi: 10.1371/journal.pone.0076131 (PMC3788761; doi:10.1371/journal.pone.0076131)
Supplement: Table S1 — Primer sequences used in qPCR. GenBank accession no. CCHamide-1, NM_001104314; CCHamide-2, NM_142028; CCHamide-1 receptor, NM_137397; CCHamide-2 receptor, NM_136355; RNApolII, NM_057358.3; RpL32, NM_170460; RpL11, NM_057706.4. (PDF) [file pone.0076131.s003.pdf]

Table S1. Primer sequences used in qPCR. GenBank accession no. CCHa 1, NM_001104314; CCHa 2, NM_142028; CCHa 1 receptor, NM_137397; CCHa 2 receptor, AY282787; RNApolII, NM_057358.3; RpL32, NM_170460; RpL11, NM_057706.4 .

**Gene**	**Direction**	**Sequence 5'-3'**	**Tm**	**Nucleotide position**
CCHa1	Sense	ACTGACGTCGGACAATTTGC	57.3 ^{o}C	126-146
	Anti-sense	ACACGAATGTCCGTATTCCA	55.2 ^{o}C	249-269
CCHa2	Sense	AAACAGCAACAGCAGCAAAC	55.2 ^{o}C	21-42
	Anti-sense	AGGACCACGGTGCAGATAAC	59.3 ^{o}C	146-166
CCHa1 receptor	Sense	GTTCCAAACACCTACATTTTATCAC	58 ^{o}C	343-368
	Anti-sense	CGGATAATGCAGTCAGCGTA	57.3 ^{o}C	490-510
CCHa2 receptor	Sense	CATACCCAACACATACATTCTTTC	57.6 ^{o}C	807-831
	Anti-sense	GAAAGGGCGGTCAGTGTAAA	57.3 ^{o}C	953-973
RNApolII	Sense	GAGATCCATCCAGCCATGAT	57.3 ^{o}C	2249-2269
	Anti-sense	TGTTACGCGGACTCTGATTG	57.3 ^{o}C	2307-2327
RpL32	Sense	CAAGAAGCTAGCCCAACCTG	59.3 ^{o}C	232-251
	Anti-sense	ACGTTGTGCACCAGGAACTT	57.3 ^{o}C	481-500
RpL11	Sense	CGATCCCTCCATCGGTATCT	59.3 ^{o}C	442-461
	Anti-sense	AACCACTTCATGGCATCCTC	57.3 ^{o}C	563-582
