# Supplementary material for: Expression Patterns of the Drosophila Neuropeptide CCHamide-2 and Its Receptor May Suggest Hormonal Signaling from the Gut to the Brain
Source: PLoS One. 2013 Oct 2;8(10):e76131. doi: 10.1371/journal.pone.0076131 (PMC3788761; doi:10.1371/journal.pone.0076131)
Supplement: Table S2 — Primers used for in situ hybridization. (PDF) [file pone.0076131.s004.pdf]

Table S2. Primers used for *in situ* hybridization

Gene or vector	Forward primer	Reverse primer	Accession number	Position of amplicon
CCHamide-1	AGGCGGTCATAGATGCTAA	TGCAATGATCTCGCGTGT	NM_001104314	301-839
CCHamide-2	AGGAACCGGACTATGTGC	CGCTGTCTTTGCCGTTTT	NM_142028	332-895
T3	ATTAACCCTCACTAAAGGGA			
T7		TAATACGACTCACTATAGGG		
